# Natural Nitrated Mineral Waters of New England

**Published:** 1914-09

**Authors:** Felix Von Oefele

**Affiliations:** 326 East 58th Street, New York City


### Natural Nitrated Mineral Waters of New England.

By DR. FELIX VON OEFELE.

A large series of sulphur springs is situated in an almost straight line from Albany to Buffalo. The development of these springs must have the highest interest to the reader of Buffalo Medical Journal. Many sick people, now travelling to European Spas, may get better therapeutic results using the mentioned home spring series. A sufficient knowledge of these American springs needs to know the relation to the springs of the neighborhood. The first chapter of this relation is the consideration of the Mineral Waters of New England.

The continuation of the Buffalo-Albany line is the Albany-Boston line. It must be considered as a geological fault through New England containing Nitrated Mineral Waters. This New England fault is crossed by two north to south faults, they also contain Nitrated Mineral Springs.

Natural Nitrated Springs are an American specialty, the therapeutical value of which is not sufficiently recognized by the medical profession. I call Nitrated Springs such mineral springs as contain more than 2 per cent. of nitric acid, calculated to total solids.

Europe and particularly Germany have no characteristic nitrated springs; that is springs with a remarkable amount of nitrates. Bath in England contains about 1 per cent. nitrates of total solids. The richest German well I know of, is Ludwigsbrunnen in "Homburg vor der Höhe" containing only 1/25 per cent. potassium nitrate in total solids. The European rocks are almost free of nitric acid. Also the European waters of magmatic origin do not carry nitric acid or nitrates. In ancient and medieval times nitrates were artificially made in Western Asia and Asiatic India and extended to Europe.

It was obtained by putrefaction from urine as potassium nitrate and sometimes on walls as calcium nitrate. Remembering this, the European balneologists conclude, that the faint presence of nitrates in a spring proves an adulteration by surface water. Trace of nitrates is oftener present in Europe, than is known. The balneological opportunity inhibits the determination of its small amounts. But large amounts are absent in Europe.

For a long time large beds of sodium nitrate are known to be in America in the South American Andes. The amount of nitrates in the Andes far surpasses that in the caves of Ceylon, the Adriatic shore of Italy or in Africa and Teneriffe. Smaller rocks of American nitrates are known to be in Tennessee, Kentucky and on the Missouri and Crooked river.

Many other places of the Old and New World contain potassium nitrate as decomposition product in the surface earth, as far as the air can penetrate; where ammonia, water and oxygen are in contact with oxygen carriers, some nitrate develops.

The high amount of nitrates and the condition of the rocks contradict the origin of nitric acid compounds from the surface of New England. The magmatic descent is only possible. Nitric acid or its compounds ascend in geological faults. We can suppose two such faults from north to south and one from east to west in New England.

The large north to south fault of nitrate springs is curved in the northern part toward the east following the direction of the shore. The axis of this fault is marked by the following Mineral Springs. We start from the north: Oak Grove Spring, Me., (3.93); Rocky Hill Spring, Me., (2.07); Poland Springs, Me., (3.67); Granite State Spring, N. H., (7.69); Burnham Spring, Mass, (10.74); Valley Spring, Mass., (15.37); Belmont Hill Spring, Mass., (30.14); Highland Spring, Mass., (12.42); and Golding Spring, Mass., (12.38). The figures in parenthesis state the percentage of nitric acid, calculated to total solids. Not one such high percentage is known in Europe. We regret that in the southern part of Massachusetts no mineral springs have been analyzed, that we can give an idea of the further position of this fault of nitrate springs until the lower coast.

The figures show an increase of nitrates to Belmont Hill Spring, then a decrease. But exactly in the north of Belmont Hill Spring the short east to west fault of New England is located containing Katahdin Spring, Mass., (30.74); Belmont Hill Spring (Mentioned before) and Lover's Leap Spring, Mass., (20.38).

Another small north to south fault starts with Equinox Spring, Vt., (2.27) and continues to Althea Spring, Conn., (18.96), and Cherry Hill Spring, Conn., (14.95). It seems the highest percentage of nitric acid is located north of Althea Spring and not yet evident by any water analysis. If this is really located near the northern boundary of Connecticut, there would be the intersection with the above mentioned east to west fault. The continuation of this fault is the springs series Albany to Buffalo.

The springs on both sides of these faults are in New England mostly not free of nitric acid. But the percentage decreases according to the distance from the faults. On the east slope of the large fault is Hale Spring, Mass., (4.95), on the west slope El Azhar Spring, Mass., (4.15); Robbins Spring, Mass., (8.64); Simpson Spring, Mass., (4.91); and Gladstone Spring, R. I., (3.15). The percentage of nitric acid is still high in these springs, because they are in the vicinity of the intersection of two faults.

My fault theory of the nitrate springs of New England has a parallelism in the theory of Gurn Spring, Saratoga Springs and Ballston Spa by Cushing and Ruedemann.

The above mentioned springs are very low mineralized. They belong to the first group of Akratopeges. Such springs have a repudiate medical value. The increase of medical value is very high on account of the oxidizing power of nitric acid and is without any European competition. Research work on pharmakotherapy of nitrates by the late professors Henoch and Frerichs in Berlin indicates good results in certain diseases of the liver, partly so in desperate diseases as cirrhosis of the liver. Especially the diuretic effect of nitrates may also aid in such cases. Besides this, nitrate preparations have been, for a long time, successfully used for asthma. The characteristic oxidizing properties of nitric acid may also increase the oxidation in the body and lead to more indications especially for aged people.

It seems therefore very important that so many nitrate springs exist in the New England states. But as yet no remarkable research work has been done in a balneotherapeutic or in a geologic or in a pharmacological medical practical line.

326 East 58th Street, New York City.

---

**Kubisagra or Gerlier's Disease.** L. P. Couchoud, Rev. de Med., April, 1914. This occurs only on the Franco-Swiss frontier and in the north of Japan. It was described independently by Gerlier and Nakano in 1844. It is a summer disease, occurring paroxysmally, usually while the patient is at work. There is weakness of the hands and arms, severe pain in the cervical region, the head falls forward and the eye lids droop. The gait is reeling and the patient may fall. There may be transitory failure of vision or diplopia. The cat is subject to the disease, and Couchoud has used it in experimental work. He claims to have demonstrated a staphylococcus, gram-negative, not liquifying gelatin, feebly agglutinated by the serum of patients and inducing the disease in cats, though not pathogenic to mice or frogs. He terms it the Micrococcus paralyticans. The prognosis of the disease is good, attacks ceasing with the advent of cool weather. (Note: Similar symptoms occur commonly from overwork, mild isolation, especially in poorly nourished subjects with mild sepsis from cuts and scratches or intestinal saprophytosis. Staphylococci can be ''isolated'' from almost everyone. Unless the bacteriologic identification is absolute, we are septic as to the regional limitation of a specific disease to two widely different localities, from which emigration has occurred extensively.—Ed.)

